# Lectures on the Exploration and Treatment of Diseases of the Chest

**Published:** 1840-06-27

**Authors:** W. W. Gerhard


### MEDICAL EXAMINER.

DEVOTED TO MEDICINE, SURGERY, AND THE COLLATERAL SCIENCES.

No. 26.] PHILADELPHIA, SATURDAY, JUNE 27, 1840. [Vol. III.

### LECTURES ON THE EXPLORATION AND TREATMENT OF DISEASES OF THE CHEST.

BY W. W. GERHARD, M. D.

### Lecture VII.—*Pleurisy.*

We now come to the study of individual diseases of the chest. These may occur in the substance of the lungs and heart, or in their investing or lining membrane. The affections of the upper portion of the respiratory system are also closely connected with those of the thorax, and will require at least a passing notice.

Both lungs and heart offer an investing and a lining membrane, which are more frequently inflamed, or otherwise affected, in connection with the parenchyma, than separately. Nevertheless, these inflammations are sometimes met with in an isolated form, and it is then that they are most readily studied; afterwards the more frequent, but more complicated forms, may be analyzed, and you may separate the symptoms belonging to the different parts. The study of special diseases may begin with the inflammation of the serous or of the mucous membranes. In the present lecture I have determined upon deviating somewhat from the ordinary course, and shall commence the study by the examination of the serous membranes. These are the most simple of all the tissues composing the lungs, and the symptoms of many of their diseases are nearly as regular and readily learned as their pathological lesions. Still, in all such cases, you must beware of the difficulty into which an imperfect study of the subject may sometimes lead you; for in diseases of the chest, more than in any others, a partial analysis, and a limited diagnosis, may become the sources of error. You must prosecute your examination until you have arrived at the knowledge of all the symptoms; otherwise the physical signs may limit your views, instead of extending them, and you may rest satisfied with the discovery of a single disorder, instead of taking into your estimate the numerous diseases with which it may be complicated. This is a common error with those who are commencing the study of auscultation; they are apt to be too well satisfied with a partial discovery of the symptoms, and to forget that many other things may be concealed which a more thorough examination would explain.

Pleurisy, as you well know, is an inflammation of the serous membrane involving the lungs; it is very regular in its progress and symptoms. Like the other inflammations of this tissue, it is sometimes simple and readily diagnosticated, and at other times is singularly complex, or perhaps consecutive to other disorders of a different and more constitutional character. For example, it may be connected with tuberculous diseases in several ways: first, tubercle may be developed in the adherent and more cellular portion of the serous membrane, and the inflammation may directly coincide with this development; in these cases the tuberculous deposit is formed, as it were, by the same process as the inflammation, and apparently by the same action of the vessels. In other cases the pleurisy is consecutive to the tubercles already formed in the lungs; in a third variety the pleurisy may attack an individual in good health, and afterwards give rise to the tuberculous deposit, partly from the general shock given to the constitution, and partly from the determination of the diseased action towards the lungs. This latter variety usually occurs in persons of a tuberculous tendency; but it may also prove a purely accidental cause of tubercles, and take place in those whose constitution is not previously tainted by this diathesis. Pleurisy also occurs in a more acute form as a complication of affections of the parenchyma of the lungs, when the latter approach the surface of the organ invested by the pleura. Pneumonia is the disease of the lungs which most frequently gives rise to this form. There are some other lesions producing the same effect, which are, however, of rare occurrence, viz., gangrene and scirrhus: when these approach the surface of the lung, they cause inflammation of the serous membrane, with an effusion of lymph,—this inflammation being in almost all cases preservative, as the adhesion which takes place prevents an effusion of the morbid matter into the cavity of the pleura.

We have, then, three principal varieties of pleurisy,—1st, simple pleurisy; 2dly, pleurisy complicated with a deposition of tubercular matter; 3dly, pleurisy complicated with acute lesion of the parenchyma of the lungs.

The pathological changes connected with ordinary pleurisy are regular in their progress, and proceed, step by step, with the symptoms, which afford us a means of measuring the intensity of the inflammation.

The first change which takes place is the injection of the membrane, connected with an enlargement of its vessels, which, in the natural state, do not transmit the red globules of the blood. These vessels are situated in the subjacent cellular tissue, and are disposed in an immense number of branches, which are interlocked in various directions, and form a complete net-work. In the midst of this, there are numerous bright red points, apparently formed by minute extravasations of blood from the vessels.

Almost simultaneous with this increase of vascularity is the development and effusion of lymph. This is at first deposited on the serous surface in minute points, which are scarcely visible, but may be readily detected by the touch. These points, as they become more numerous, gradually collect into groups, which, finally coalescing, form a continuous membrane. This deposit of lymph has received the name of a false membrane, and is more abundant at the lower portions, where it is in some cases as much as a fourth or even half of an inch in thickness, while at the upper portion it seldom exceeds the eighth of an inch. The character and amount of the effusion vary according to the form of the disease, and the constitution of the individual affected. In cases of local pleurisy, especially if occurring in robust persons, the amount of serum effused is very small, while there is a considerable deposit of lymph; the same also occurs in persons who are not robust when the inflammation is confined to a small portion of the membrane. On the contrary, if the patient be thin, and of a lymphatic temperament, and the inflammation diffused, the effusion of serum will be very great, with but a slight trace of lymph. The thin and serous part of the effusion tends to diffuse itself over the surface of the pleura, gravitating to the most dependent portion, and shifting its position with the movement of the patient. When, however, it is principally composed of lymph, it is confined to the part of the lung which is affected, and exhibits no such tendency. The serum increases in quantity as the disease advances, and decreases with its decline; but the lymph is more persistent in character, and, instead of being removed, becomes organized, and assumes the character of a serous or cellular membrane, according to the circumstances in which it is placed. When the inflammation continues for a considerable length of time, a secretion of pus takes place, and the serum is entirely replaced by purulent matter. The lymph in this case being bathed in pus, is modified in colour by its assuming a yellowish hue. When the serum is abundant, the lower portion of it is turbid, while the upper portion is clear. This results from the greater specific gravity of the lymph, in consequence of which it settles to the bottom of the fluid.

During the recovery of the patient, the following changes are observed to take place. As the serum is absorbed, the pressure of the atmosphere forces the parietes of the chest towards the lung, and adhesion takes place between the two surfaces of the pleura. As the lung is compressed against the spine, and in that position is covered with a coating of lymph, it remains permanently flattened, and cannot rise to meet the ribs. In those cases in which the pleurisy is slight, and the effusion very small, there is either no contraction of the chest, or it takes place to a very slight degree. The contraction is not entirely permanent; the lung after being compressed, does again expand to a certain extent, and rises partially towards its original form.

The adhesions become gradually organized during this process, and new vessels are formed in the lymph. The particles of blood are deposited in the lymph under form of dots, and gradually collect in trains or streaks; vessels are afterwards formed around the blood, which then finally inosculate with the original vessels of the subjacent serous tissue. The contraction of the chest is not great when the serum is but moderate in quantity; but in cases of abundant effusion, the contraction is equally well marked with the previous distension. The alteration of conformation, therefore, is a purely pathological state, and corresponds accurately with the quantity of liquid exhaled. If, therefore, the effusion be limited, it does not produce a very decided dilatation or subsequent contraction: a less quantity than a pint is scarcely appreciable; a quart gives rise to a very decided alteration in the shape of the chest, and larger quantities distend it sufficiently to incline the body towards the sound side. In the same way, if the contraction which follows pleurisy be very great, the body is inclined towards the diseased side.

The nature of the liquid is not always the same; the greatest portion of it consists of serum in the early or inflammatory conditions of the disease. This is mingled with flocculi of lymph of various density, which seem to be detached from the surface of the pleura. In the chronic varieties of the disease, the liquid consists almost exclusively of purulent matter, although at first the serum is merely tinged with pus from a small admixture of globules with it; but as the disease continues, the purulent globules become gradually more and more abundant, until the liquid consists nearly of pure pus: the pleurisy is then often called empyema. It is in these cases that the distension of the chest is greatest. In the early stages of some cases, pus is mixed with the serum and lymph in small quantity, giving the liquid a slightly yellowish tinge; but, as a general rule, it is quite transparent, but of a light greenish yellow colour. In a few instances it coagulates spontaneously immediately after death, becoming a mass of tolerably dense albumen. In a number of cases it contains blood in small quantities, and occasionally, although rarely, the proportion of blood is large. These varieties in the exhaled fluid belong to the same disorder, which is in all these cases inflammatory; but the product varies according to the general condition of the individual's previous health, and other circumstances difficult to discover. In general, the product of inflammation of the pleura, and other serous membranes, is most consistent and most highly animalized when the patient is strongest, and the disease most violent.

These changes are very regular, and give rise to an equally regular succession in the physical signs. When the inflammation is severe, and the effusions very large, these signs are pathognomonic of the disease; but when it is small, the physical characters are so far useful, that they either confirm the indications of the functional signs, or prove that the disease is not advanced beyond a certain point. When the effusion of serum takes place, the sound on percussion is immediately dull, becoming gradually flat as the quantity of the liquid increases. The flatness is much more decided at the lower than at the upper portion of the chest, and becomes gradually less in ascending towards the summit; for the liquid of course gravitates towards the most depending portions. Still, the serous effusion is not the only cause of the flatness; it depends, in part, upon the thick deposits of lymph at the inferior portion of the lungs, and does not disappear entirely when the position of the patient is changed, although a change in the level of the liquid is always attended by a change in the degree of flatness. If the effusion be very large, the flatness gradually becomes more complete, and at the same time extends over the side of the chest, until the resonance is either completely lost, or is limited to a small portion of the chest near the spine, where the lung generally contains a little air. The increase in the flatness enables us to estimate the extent of the effusion with great accuracy; but the converse of this is not true in its declining stage,—for when the compression of the lung is carried to a great extent, it recovers its elasticity but slowly, and remains either permanently or for a long period in a more solid state than is natural; hence the clear sound returns slowly, and generally never recovers its original sonorousness. A moderate but diffused resonance does not, therefore, prove that the lung has not recovered from the inflammation.

The enlargement of the affected side accords with the dulness on percussion, and is always met with when the dull sound is at all decided. If, in the early stages of the pleurisy, you examine the lower and posterior parts of the chest, you will readily detect slight changes in the conformation; and this is then generally limited to an alteration of the natural convexity of the thorax, and is scarcely perceptible in the whole semi-circumference. The quantity of liquid which is sufficient to cause a decided change in the conformation, varies from a pint to several gallons. When it exceeds a gallon, the distension is of course very great. I have, on one occasion, in which the bulging of the affected side was immense, found no less than five gallons in the right pleura. In these extreme cases the healthy lung is compressed towards the ribs, at the same time that the diseased one is forced against the spine, and death usually occurs from suffocation. The semi-circumference of the chest may be measured with a tape on a level with the sixth or seventh dorsal vertebra, in order to give you an idea of the changes which take place in the quantity of the liquid; but this method is of little use except in cases in which the effusion is very large. The position of the heart is another sign which is closely connected with the alteration in the conformation. If the pleurisy occur on the left side, the heart is sometimes forced to the right of the sternum; if, as is most frequent, the pleurisy attack the right side, the heart is removed towards the left axilla.

The respiration in the early stages of pleurisy is always feeble,—that is, if either the pain is tolerably acute, or the effusion at all considerable. But at the beginning, the feebleness depends much more upon the pain than the mechanical pressure of an effusion which is still quite small in quantity. When the dilatation of the vesicles in a part of the chest is attended with pain, that portion of the lung becomes to a great degree motionless, and remains so until the pain diminishes. This rule is so general in its application, that if the serous membranes of the chest be inflamed, in a great extent, and over both lungs, the patient may perish from the dyspnœa which arises from the inactivity of so large a portion of the pulmonary tissue. The feebleness of the respiration continues throughout the disease in those portions of the lungs in which the bronchial tubes are small; where they are much larger, the respiration becomes more or less bronchial, or at least rude. The intensity of the rude respiration varies very much, and chiefly according to the condensation of the lung; when this is very great, the bronchial respiration is very intense, sometimes quite as loud as in the most severe cases of pneumonia. The condensation of the substance of the lung is, therefore, a circumstance which favours the bronchial respiration. The density of the effused liquid is another cause of the loudness of the bronchial respiration; if there be a large proportion of lymph, or a thick, viscid liquid in place of the usual thin serum, the conducting power of the substance which intervenes between the tubes and your ear is increased, and the same result is produced as if the lung itself were inflamed.

When there is bronchial respiration in pleurisy, the resonance of the voice becomes bronchial, and you will observe a true bronchophony. This has, however, a peculiar vibration or quivering in its tone, which never exists to the same degree in pneumonia proper. If the bronchial respiration is not so loud, the resonance of the voice becomes less bronchial, but its vibration is increased, and its resonance is termed egophony. This takes place in those cases in which the effusion is but of moderate density, or little more thick than ordinary serum; and it is heard most distinctly from the anterior portion of the axilla to the scapula, and between this bone and the spine. It is therefore most evident when the bronchial tubes are moderately large, and there is a tolerably strong compression upon the vesicles. The depth of tone of egophony is modified by the density of the liquid more than its vibration; if the liquid remain thin, the egophony will continue; but in proportion as the density of the lung and of the effused fluid approaches more nearly to that of pneumonia, the resonance becomes more like bronchophony than egophony. When the egophony is perfectly pure, it is less loud, and often less easily recognised than in those cases in which the body of sound is decidedly increased by the hardness of the lung.

In certain cases of pleurisy there is little resonance and no vibration of the voice; this must depend upon the obstructions which prevent the passage of the air through the tubes, and of course destroy the resonance. It is difficult to state what these obstructions are; in some cases they may depend upon the pressure of the liquid upon the tubes, in such a manner as to interrupt the column of air, or upon accidental collections of liquid in them. If the lung remain soft and uncompressed, it will also give rise to an egophony which is but moderately loud; the circumstances, therefore, which favour its development, are moderate pressure and a little increase in the density of the tissue of the lung. If the voice be shrill and clear, it is of course much more decided.

The friction sound is another sign of pleurisy, which is much more irregular than the resonance of the voice. It occurs under two different circumstances, at the beginning and towards the termination of the disease,—that is, at those times in which the effused matter consists almost exclusively of lymph, and not of serum; for if there be a large and thin effusion, the friction of the two surfaces of the pleura, which is the essential cause of this sound, will be prevented. When this sound occurs early in the disease, it of course takes place in the variety of pleurisy which may be termed dry, whether it continue in that stage or not; the friction is then very slight, and is unappreciable by many persons; it is more like the slight noise produced by rubbing together two pieces of tissue paper than any thing else. When it occurs at the close of the disease, after the absorption of the liquid, it is much louder, and then offers the peculiar character of the true friction sound. This is sometimes quite permanent, lasting several minutes, or even much longer. These irregular sounds are not of value for the proper diagnosis of pleurisy; they are only of accessary importance, and should be recollected by you, because every thing should be known which may become of use under any circumstances.

The signs of the lungs, properly speaking, are of great negative importance in the diagnosis of pleurisy. In fact, it is at times impossible to distinguish the cases in which the lung is unaffected, in any other way. If, therefore, you find no signs of pulmonary disease, such as are indicated by the rhonchi and respiration, you may regard the case as one of simple pleurisy. But, in order to form this opinion, you must take into your calculation both the general and local signs of pulmonary disease; and even then it will stand good only for the time, for you may be afterwards obliged to modify your opinion. Still, in simple pleurisy, you should recollect that there are no signs of disease of the lungs, other than those which arise from their consolidation by the pressure of the liquid. In practice, the complicated cases are probably quite as frequent as those which are more simple.

In the recovery from pleurisy, restoration to health takes place but slowly, and the lung does not recover its natural respiration for a considerable time; the sound remains feeble, and the percussion dull: after a very long period, sometimes a year or more, the restoration to the natural fulness and softness of the respiration may take place; but this is not to be anticipated in the great majority of cases, and we must therefore be satisfied with a slow and gradual improvement.

Besides the physical signs, there are other symptoms of pleurisy, which are, to a certain extent, quite conclusive. These are generally most decided in the commencing stages of the disease, and they may subside almost entirely, and be almost forgotten by the patient. The diagnosis of the disease is therefore easiest, by the general symptoms, at its very commencement, when the physical signs are most obscure. We are also obliged to rely chiefly upon the rational symptoms in those cases in which the adhesions between the two surfaces of the pleuræ are strong, and of course no effusion can take place; this is always the case in pleurisy which has succeeded to a former severe attack of the same disease.

Of these local, but at the same time functional signs, the most prominent is the pain. This is so acute in many cases of pleurisy, that the ideas of pain and pleurisy are very firmly associated in the minds of most persons, and they are apt to believe that all cases of pleurisy must be attended with pain: this is an error; for the pain may either be totally absent, or so obscure as scarcely to attract attention; it is then limited to a mere soreness along the portion of the chest most affected. When there is severe pain, it is almost always felt near the nipple; it is acute and lancinating, similar to that caused by the prick of some sharp instrument; hence it is in many languages called a stitch in the side. It is increased by motion, cough, or even respiration. When the inflammation is very sudden and extensive, the pain may be agonizing, and for a time effectually check the respiration. A large quantity of effused liquid rather diminishes than increases the pain; and when it becomes very large, as in very chronic cases, the pain is often limited to a mere soreness, which is often seated in the loins, instead of the thorax. This seems to depend upon the great weight of the thick purulent liquid. In diaphragmatic pleurisy, especially when caused by rheumatic or gouty disease, the pain is difficult to localize, and is generally wandering about the lower part of the thorax, causing more distress than other varieties of the disease. You perceive, therefore, that the pain is an important symptom of the disease when it exists, but that it is never lasting in the slow and moderately severe cases of pleurisy, and may be either entirely absent or badly characterized throughout the disease.

The cough is another local symptom: this is generally present in the milder cases of the disease, and is always short and almost insignificant. If the inflammation be very acute, the cough is almost entirely suppressed; and even in moderately severe cases, it is in a great degree checked by the aversion of the patient to make the strong respiratory movement necessary to produce a full cough. It is not attended with expectoration in the simple inflammation of the pleura, for there is of course no secretion to be thrown off externally, unless the substance of the lung or the bronchial tubes are involved in the disease. Hence many of the remarks which you will find in some of the older writers upon this subject, are in reality applicable to pneumonia, and not to pleurisy. The more chronic the disease becomes, the less disposition is usually felt to cough, and in cases of extensive empyema, there is often no cough.

The mode in which the respiration is performed is sometimes of importance; in the beginning of the disease, when the pain is severe, the patient breathes chiefly with the healthy lung: this arises from the pain which is caused by the act of respiration, as well as coughing. When the disease is more advanced, the mechanical pressure upon the affected lung will prevent its expansion. Hence the patient throughout the disease breathes chiefly by the healthy side.

The decubitus in pleurisy is sometimes of importance. When there is pain, you may state, in general terms, that the patient does not lie upon the affected side, which is extremely sensitive to pressure. Even late in the disease, he will prefer the sound side, or the back; but when the effusion is so great that the weight of the liquid would press upon the mediastinum, and thus prevent the expansion of the healthy lung, he will naturally prefer lying upon the diseased side, and will thus relieve the lung which remains in a state fit for the performance of its proper functions.

The rational as well as physical signs which I have just described, are those which belong to pleurisy considered chiefly as a local affection. There are many other symptoms which appertain to it in common with other inflammations of the serous tissues. These phlegmasiæ present a number of characters similar to those of other inflammatory affections, and some that are nearly peculiar to themselves. In general, the serous tissues, like the rest of the body, modify the ordinary characters of inflammation, rather than offer others which are strictly novel.

At the commencement there is usually a chill, which varies in intensity from a slight sensation of coldness to a complete chill. This is generally felt at the same time with the pain,—that is, the pain in the chest excites the chill; it may return at several different times throughout the disease; but it then rarely offers the same intensity as on the first day. The chill is followed, of course, by heat, and by sweating, which occurs at irregular times, and is never very copious. During the disease the fever is generally persistent, and is characterized by a quick, tense, but rather small pulse. This is often called the pulse of inflammation of the serous tissues; although not regularly present in all cases of these diseases, it is found in a large proportion of them. The sweats in pleurisy are sometimes extremely abundant, especially in the varieties of the disease that are complicated with a tuberculous development; but even in simple inflammation of the pleura they are sometimes extremely copious, and form a harassing and alarming symptom. In empyema, the nature of the fever approaches the hectic type, and almost always assumes it when the operation of paracentesis has been performed, and a free communication is made between the external air and the purulent collection. In the latent form of pleurisy the fever may be quite moderate, rather a slow febricula than a perfect fever, and this is one of the causes which render this form of the disease extremely obscure.

The secondary irritation and inflammation of other viscera, which are so frequent in the inflammations of the mucous membranes and the parenchymatous organs, are very slight in pleurisy and serous inflammations in general. The disturbance of the alimentary canal is strictly proportioned to the intensity of the fever, and not to the gravity of the inflammation, which pursues a course almost unconnected with the other viscera. The strength and the cerebral functions are usually just so far affected, as naturally results from the severity of the pain and degree of the fever; they are, in themselves, very little disturbed by the inflamed pleura. Hence pleurisy is a remarkably simple disease, if it be the primary affection; it forms itself the complication, but has little power to give rise to disorder of other tissues. This is explicable enough when you reflect upon the simple structure and few nervous relations of the serous tissues. There is, however, one exception, the tuberculous diseases, whose development is sometimes singularly favoured by pleurisy.

The diagnosis of pleurisy is readily enough made in most instances: a well characterized case is always certainly known, and can be confounded with no other affection. That is, when the distension of the chest, the dulness on percussion, and feeble or bronchial respiration, coincide with dyspnœa, pain, and fever. If you restrict your diagnosis to the functional signs, you will, of course, be somewhat puzzled in many cases: with the aid of the physical signs, all decided cases can be mistaken for nothing else. In the slighter cases, where there is little or no physical change, this is not always the case: pleurisy may be confounded with pleurodynia, or simple rheumatic pain in the intercostal muscles and the adjacent fibrous tissues. The fever is a very uncertain test; but it has a collateral value, for it is more apt to accompany true pleurisy than simple pleurodynia. The nature of the pain is a better one; for, in pleurisy, this is, to a certain degree, limited, and almost always is found about the anterior margin of the axilla; but in pleurodynia it shifts about, and is often found on both sides at once; very frequently it disappears for a time, but soon returns, displaying in this respect the peculiar changeable character of rheumatic disease. When the pain occurs during the course of inflammatory rheumatism, you need not trouble yourselves about the diagnosis,—for in such cases there is almost always something more than a mere rheumatic pain, and the pleura is positively, though perhaps slightly, inflamed. As a general rule, therefore, if in your suspected pleurisy the pain is at all constant, you may regard it as a true inflammation. The mobility of the pain is therefore the only good proof of pleurodynia. There is no difficulty in distinguishing between simple pleurisy and pneumonia, or other diseases of the parenchyma of the lungs, with pleuritic complications; for the signs of true pulmonary disease are of course wanting in the one case, but present in the other. The inflammation of the pericardium frequently occurs in connection with pleurisy of the left side, when it is sometimes extremely difficult to recognise it; for the signs of one disease, to a great extent, obscure those of the other. If the pleurisy attack the right side, the distinctive characters of the two diseases are quite evident.

In simple pleurisy your prognosis is almost always favourable if you see the patient rather early in the disease; if the effusion is very large, or if the disease be chronic, it is then quite doubtful: the mortality is totally different under these circumstances. In the secondary pleurisy, or in that variety which is accompanied by tuberculous disease, the prognosis is of course much less favourable. When it precedes tubercles, it usually ends in recovery, but afterwards gives rise to them.

The treatment of ordinary pleurisy is based upon well established grounds,—that is, of the disease as distinguished from those cases in which pneumonia plays the most important part. It is strictly antiphlogistic,—and, as in other inflammations of the serous membranes near the surface of the body, is most effectual when you use local depletion in combination with or in addition to general blood-letting. The latter remedy, however, is always productive of great relief in the cases which begin with strong inflammatory symptoms,—that is, much pain and dyspnœa; there is, then, no substitute for it. After you have taken a moderate quantity of blood, however, and have relieved the pressing symptoms, the indications are then rather to continue the treatment by local depletion and by diaphoretics than repeated general bleeding. Cupping or leeching to the painful parts, repeated if necessary, two or three times, is then the best remedy. The effects of local bleeding are much more prompt in serous than in mucous inflammations, or in diseases of the parenchyma of organs. They may be repeatedly applied in either acute or chronic cases; but you will gain most from them if you choose the moment when the pain is most acute; it will then often yield very quickly, and the disease improve after free local bleeding.

There are several other local remedies which are effectual in relieving the pain and inflammation besides cupping and leeching; these are warm poultices of hops, sprinkled with a teaspoonful or two of laudanum, and kept warm by placing over them a bottle or tin vessel filled with hot water, which should lie on the bed by the side of the patient. The narcotic acts with considerable energy upon the part, and its action is favoured by the warmth and moisture. In slight pleuritic pains, as well as in the true pleurodynia, sinapisms are eminently useful; but they are of little benefit in severe pleurisy. This is not the case with blisters, which belong to that established class of remedies whose virtues have been tested by the experience of many generations; they are used with two objects in view—to relieve the inflammation, and to favour the absorption: hence they are particularly applicable to those cases in which there is much effusion, both in the acute and chronic diseases; while the inflammation is still advancing, the operation of blisters is uncertain, and sometimes seems to be positively injurious; but after the active inflammatory symptoms have been checked, they are productive of decided benefit, and are, perhaps, of all remedies, those whose action is most unquestionable, as the acute pain often subsides immediately after vesication, and the absorption of the effused liquid sometimes takes place very rapidly. The rapidity of absorption is not generally proportioned to the quantity of the serous secretion from the blister, although in a few cases a very copious discharge will pour from the vesicated surface, and the pleuritic effusion will disappear in a few hours. In chronic cases of pleurisy, blisters are amongst our most valuable remedies; but they should be small, and very frequently repeated. My own plan is not to make them larger than two or three inches square, and to apply them every two or three days, dressing the surfaces with simple cerate; you should, in this way, pass over a considerable part of the affected side by applying these small blisters successively to different parts of it.

When the pleurisy has been entirely or nearly removed, the patient often complains of slight returns of the pain from exposure to damp, or to a cold wind. The only way of guarding against these slight returns of the inflammation is to cover the affected side with a Burgundy pitch plaster, and to direct your patient to clothe himself warmly.

These, then, are the directly depletory remedies, and such as act as local counter-irritants. The internal remedies suited for the treatment of pleurisy are numerous, and applicable either to different cases of the disease, or different stages of the same affection. They may be divided into three principal classes:—1st. The antiphlogistic remedies, which are intended to relieve the inflammation, and check the fever. 2d. The remedies that promote absorption, which, however, are often fitted at the same time to check the inflammation. 3d. The anodyne, which may relieve the pain. Of course, in a strictly inflammatory disease, the first class of remedies, and those which belong both to the first and second classes, are the most important. The tartarized antimony has long been used both in simple pleurisy, and in the disease complicated with pneumonia; it is usually given as a diaphoretic, in the doses of a fourth to the eighth of a grain,—rarely in larger doses. In these doses its nauseating influence is but slight. In the early stages of pleurisy, free diaphoresis is a powerful therapeutic agent,—but in the more advanced cases, sweating is productive of comparatively little benefit: its good effects seem to be limited to those stages of the disease in which resolution is practicable before there is much effusion into the pleura; that is, it is a means of depletion from the vessels, and exercises comparatively little influence in promoting absorption. In chronic cases of pleurisy the tartar emetic should either be given up altogether, or restricted to very minute or merely alterative doses.

The tartar emetic is almost the only remedy which is nearly exclusively antiphlogistic in its action; most other internal remedies are more powerful from a combined action in promoting absorption, and checking inflammation. The most important are mercury, nitre, and digitalis; squill and colchicum are also powerful remedies, and act like most other diuretics of a moderately stimulating character. Of these the most important is mercury. Mercury, given in moderate doses, so as not to disorder the bowels, produces two distinct effects; one is directly antiphlogistic, the other is the influence which it exerts upon secretion and absorption. In the treatment of pleurisy in its active inflammatory stage, the first action of mercury is that which is most beneficial; in the advanced cases of purulent effusions, the inflammatory character of the disease is less marked, and the action of the mercury is chiefly limited to the absorption and elimination of the effused matter from the body. In the more acute cases you may give mercury more rapidly, in chronic cases more slowly. Thus, I would advise a quarter of a grain to half a grain of calomel to be given every four hours, if you design it as an antiphlogistic; it will then produce its specific effect in a short time, and the disease will generally decline. The mercurial treatment is of course but a sorry substitute for bloodletting, which it should follow and assist, but not replace. If the mercury be used towards the decline, or in the advanced periods of the disease, when your object is more to promote absorption than to remove the inflammation, you should give the calomel in much smaller doses, as an eighth or a sixth of a grain three or four times a day: this operates but slowly, and is much more effectual in increasing the power of other alteratives than larger quantities.

The mercurials are usually combined with other remedies, which will work, as it were, in the same direction with them. Thus, in the early stages of the disease, Dover's powder, or the simple opium and ipecacuanha, may be given with them: if full diaphoresis is brought about by these means, the disease is more easily subdued. In the advanced stages, digitalis, and nitre, act admirably as diuretics. There are cases in which others of a more stimulating kind, as the juniper berries, or spirit of nitre, come in well; but these are chiefly such cases as approach very nearly to hydrothorax: there is then a feeble condition of the economy, and but little active inflammation.

The diaphoretics of a vegetable kind are, like many other remedies, adapted for various stages of pleurisy. In the early stages, full diaphoresis acts admirably as an antiphlogistic remedy, while in the advanced stages it may increase absorption, and remove the effused fluid. The latter effect is, however, very uncertain; for the disease naturally tends to produce sweating, and the perspiration seems an abortive attempt on the part of nature to throw off the disorder,—the curative action being quite disproportioned to the diseased one.

Anodyne remedies in the treatment of simple pleurisy, are merely palliative, and are, therefore, rarely given alone. They consist almost entirely of some form of opium, except in those cases in which the patient is unable to take any preparation of this drug: we are then compelled to resort to various substitutes. You must not, however, suppose that opium is insignificant, or of no value, because it is simply a palliative; for in pleurisy, as in other inflammations, the relief of pain prevents the increase of the disease, and is one of the most effectual aids towards its cure. The only objections to its employment are to be found in those cases in which the cure takes place chiefly by secretions which must be thrown off from the body: this is not the case in inflammation of the serous tissues, in which the liquid is necessarily retained until it can be removed by absorption and the adhesion of the coagulable lymph. There is, then, no permanent therapeutic contra-indication to the use of opiates: if the skin be dry, they should be given in the form of Dover's powders, from eight to twelve grains of which may be given in divided doses during the day. If the sweating be copious, morphine will, as a general rule, be the best remedy, administered chiefly at night, in the ordinary doses of an eighth to a quarter of a grain. This is sometimes necessary for a considerable period.

When you find the pleurisy nearly well, but the patient still complaining of some dyspnœa, or a little feverishness, and you discover, on examination, that a portion of the liquid remains unabsorbed, nothing is so efficacious as a journey, with its necessary consequence, change of air. Although the sea-air is not always adapted to pectoral diseases, it is often of decided advantage in chronic pleurisy, especially if combined with a voyage. But a course of this kind is necessarily attended with no little expense and inconvenience, and is totally beyond the reach of many of your patients: you will be obliged to resort more frequently to land journeys, as a less troublesome and sometimes as efficient a course. This is generally the surest means of dissipating the remains of the disease, and insuring a restoration to entire health. Of course, the usual hygienic precautions as to dress, should be adhered to.

There is no disease in which the treatment is more influenced by a knowledge of its symptoms and pathological relations than pleurisy; for, simple as it is, the success, in chronic cases, depends chiefly upon steadily watching the physical condition of the chest, and persevering in your care until the disease is entirely dissipated.

